# The Effects of 1α, 25-dihydroxyvitamin D_3_ and Transforming Growth Factor-β3 on Bone Development in an *Ex Vivo* Organotypic Culture System of Embryonic Chick Femora

**DOI:** 10.1371/journal.pone.0121653

**Published:** 2015-04-02

**Authors:** Emma L. Smith, Hassan Rashidi, Janos M. Kanczler, Kevin M. Shakesheff, Richard O. C. Oreffo

**Affiliations:** 1 Bone & Joint Research Group, Human Development and Health, University of Southampton, Southampton, United Kingdom; 2 Centre for Biomolecular Sciences, School of Pharmacy, University Park, University of Nottingham, Nottingham, United Kingdom; Nanjing Medical University, CHINA

## Abstract

Transforming growth factor-beta3 (TGF-β3) and 1α,25-dihydroxyvitamin D_3_ (1α,25 (OH) _2_D_3_) are essential factors in chondrogenesis and osteogenesis respectively. These factors also play a fundamental role in the developmental processes and the maintenance of skeletal integrity, but their respective direct effects on these processes are not fully understood. Using an organotypic bone rudiment culture system the current study has examined the direct roles the osteotropic factors 1α,25 (OH)_2_D_3_ and TGF-β3 exert on the development and modulation of the three dimensional structure of the embryonic femur. Isolated embryonic chick femurs (E11) were organotypically cultured for 10 days in basal media, or basal media supplemented with either 1α,25 (OH) _2_D_3_ (25 nM) or TGF-β3 (5 ng/mL & 15 ng/mL). Analyses of the femurs were undertaken using micro-computed tomography (μCT), histology and immunohistochemistry. 1α,25 (OH)2D3 supplemented cultures enhanced osteogenesis directly in the developing femurs with elevated levels of osteogenic markers such as type 1 collagen. In marked contrast organotypic femur cultures supplemented with TGF-β3 (5 ng/mL & 15 ng/mL) demonstrated enhanced chondrogenesis with a reduction in osteogenesis. These studies demonstrate the efficacy of the *ex vivo* organotypic embryonic femur culture employed to elucidate the direct roles of these molecules, 1α,25 (OH) _2_D_3_ and TGF-β3 on the structural development of embryonic bone within a three dimensional framework. We conclude that 1α,25(OH)_2_D and TGF-β3 modify directly the various cell populations in bone rudiment organotypic cultures effecting tissue metabolism resulting in significant changes in embryonic bone growth and modulation. Understanding the roles of osteotropic agents in the process of skeletal development is integral to developing new strategies for the recapitulation of bone tissue in later life.

## Introduction

1α,25-dihydroxyvitamin D_3_ (1α,25(OH)_2_D_3_) and transforming growth factor-beta3 (TGF-β3) play significant, but contrasting roles in the development, maintenance and repair of the skeleton [1–7]. The steroid hormone 1α,25(OH)_2_D_3_ (calcitriol) has been extensively characterized as an essential factor in the regulation of calcium, phosphate [8] as well as in the mineralization processes of bone [9]. 1α,25(OH)_2_D_3_ metabolites together with parathyroid hormone (PTH) have been demonstrated to elevate bone formation in early embryonic chick femurs [10]. Recent supporting evidence has determined that fetal skeletal development can be dramatically affected by the levels of maternal 1α,25(OH)_2_D_3_ expressed in early pregnancy [11] and that reduced skeletal integrity in the offspring in later life has been attributed to a reduction in critical levels of 1α,25(OH)_2_D_3_ during pregnancy [12], [13]. Historically, the most common outcome of a lack of adequate levels of 1α,25(OH)_2_D_3_ in the body is the development of clinically related orthopaedic problems such as rickets [14]. However, the direct role of 1α,25(OH)_2_D_3_ on embryonic bone formation remains to be fully elucidated.

TGF-β3, a polyfunctional regulatory cytokine of the TGF beta superfamily plays a significant role in the differentiation of mesenchymal stem cells to chondrocytes and the induction of cartilage formation both *in vitro* and *in vivo* [15–17]. TGF-β3 has been found to be highly expressed in the proliferating zone of cartilaginous growth plates [18] and in the cytoplasm in early hypertrophic chondrocytes in three week old chick bones [19]. It is an essential factor during chondrocyte differentiation in the development of the chick femur [20] and has the ability to reduce the osteogenic and mineralization processes in human mesenchymal stem cells (MSCs) [7,19]; while direct administration of TGF-β3 to rat cranial sutures can limit the proliferative capacity of osteoblasts [21]. Furthermore, inhibition of TGF-β3 receptors has been shown to result in premature ossification of the cranial suture [22–24]. In tissue engineering, TGF-β3 has been demonstrated to play a fundamental role in enhancing the chondrogenic differentiation of MSCs to develop cartilage [25]. However, there remains a paucity of information on the direct actions of TGF-β3 on osteogenic differentiation, bone formation and skeletal development.

In normal fetal bone development various cell types, bioactive factors, environmental cues, including for example mechanical forces, function within precise temporospatial mechanisms [26,27]. Understanding the effects that 1α,25(OH)_2_D_3_ and TGF-β3 exert on skeletal development using an ex vivo chick femur organotypic model may provide functional and mechanistic clues to generate new therapies for skeletal regeneration.

The current work has examined the effects of the 1α,25(OH)_2_D_3_ and TGF-β3 on skeletal development in the ex vivo chick femur organotypic model, with its multiple cell types and in situ arrangement within a natural extracellular matrix.

## Materials and Methods

### Materials

6 well tissue culture plates were obtained from Greiner BioOne, UK. Millicell culture inserts (0.4μm pore size) (Cat No. PICM03050) were purchased from Millipore (30 mm diameter). Transforming Growth Factor-β3 (TGF-β3) was purchased from Invitrogen, Scotland. Phosphate Buffered Saline (PBS) was purchased from Lonza Biologics, UK. 1α,25-dihydroxyvitamin D_3_ (1α,25(OH)_2_D_3_) and all other tissue culture reagents and media were obtained from Sigma-Aldrich, UK unless stated. LF68 Type I collagen antibody (polyclonal rabbit) was a kind gift from Larry Fisher at the NIH, Bethesda, USA. Type II collagen antibody (rabbit polyclonal) was purchased from Calbiochem, UK and proliferating cell nuclear antigen (PCNA) antibody (mouse monoclonal) was purchased from ABcam, UK. STRO-1^+^ antibody culture supernatant was obtained from the STRO-1^+^ hybridoma provided by Dr J. Beresford, University of Bath.

### Methods

#### Organotypic cultures of embryonic chick femurs

All animal procedures were carried out in accordance with the guidelines and regulations laid down in the Animals (Scientific Procedures) Act 1986. Chick embryos were sacrificed at D11 and D13 by schedule 1 decapitation according to Home Office Approval UK (Project license—PPL 30/2762). Femora were dissected from, 11- and 13-day-old chick embryos *(Gallus domesticus)*, where the soft tissue such as adherent muscles and ligaments were carefully removed while preserving the periosteum. The dissected femurs for organotypic cultures were washed in 1x PBS and placed in the organotypic set up as previously stated [28,29]. In brief, bones were transferred to six well plates and positioned resting on 0.4 μm filter well inserts at the interface between the air and the basal culture medium (1 mL of basal tissue culture medium (TCM) consisting of α-MEM, penicillin (100 U/mL), streptomycin (100 μg/mL), and ascorbic acid 2-phosphate (100 μM)). E11 femurs were organotypic cultured in basal TCM (1 mL) and basal TCM supplemented with 1α,25(OH)_2_D_3_ (25 nM). E11 and E13 femurs were organotypic cultured in basal TCM (1 mL) and basal TCM supplemented with TGF-β3 (5 ng/mL & 15 ng/mL). All femurs were organotypic cultured for a period of 10 days providing an air/liquid interface with the femur. Culture media was changed daily for the duration of the experiment (10 days) (n = 4 femurs per group). At the end of the experiments, organotypic cultured femurs were washed in 1x PBS and fixed in 4% paraformaldehyde (PFA). The femur samples were then imaged radiographically using a Faxitron Specimen Radiography System (MX-20) (Qados Ltd, Sandhurst, UK) and the lengths measured. The non-cultured and organotypic cultured femurs were then scanned using μCT and then either assessed for quantification of glycosaminoglycan (GAG) content using a dimethylmethylene blue (DMMB) colorimetric assay, or processed for histology.

### Microcomputed Tomography

Quantitative 3D analysis of non-cultured embryonic chick femurs and organotypic cultured embryonic chick femurs was performed using an Xtek BenchTop 160Xi CT scanning system for μCT (X-TEK Systems Ltd, Tring, Hertfordshire, UK) equipped with a Hamamatsu C7943 x-ray flat panel sensor (Hamamatsu Photonics, Welwyn Garden City, Hertfordshire, UK). The femur samples were centered in the middle of the μCT X-TEK machine, focused, calibrated and adjusted to prevent X-ray saturation of the sample. Femur samples were scanned using settings 60Kv, 150μA with a molybdenum target with an exposure time of 1067m/s, 2x digital gain, number of angular positions at 701 and scans performed with minimized ring artifacts. Raw data was collected and reconstructed using CTPro (X-TEK Systems Ltd, Tring, Hertfordshire, UK) with a mean 11 μm voxel resolution. The reconstructed femurs were selected for quantification of bone. The reconstructed images were visualized and analyzed using Volume Graphics (VG) Studio Max 1.2.1 software package (Volume Graphics, GmbH, and Heidelberg, Germany). After segmentation thresholds were used to remove the soft tissue, Bone Volume (BV) (mm^3^), Bone Surface/Bone Volume (BS/BV) (mm^-1^), Bone Volume/Total (tissue) Volume (BV/TV) (%), Trabecular Thickness (Tb.Th) (mm^3^), Trabecular Number (Tb.No) (mm^-1^) and Trabecular Spacing (Tb.Sp) (mm) were then calculated. 3D images were created and saved as TIFF & JPEG interchangeable files.

### Colorimetric DMMB assay for quantification of glycosaminoglycan content in the organotypic cultured femurs

Following fixation in PFA, femurs were weighed and digested in 1.06mg/ml papain solution (1 mL per femur) overnight at 60°C. 20 μl of each digested femur sample was pipetted into a 96 well tissue culture plate (Greiner BioOne, UK) and 200μL of dimethylmethylene blue (DMMB) solution (Sigma, UK) added to each well. 20μL of standards of known concentrations of chondroitin-4-sulphate salt from shark cartilage (Sigma, UK) between 0 μg/mL and 100 μg/mL were also added to 200 μL DMMB solution, to calculate a standard curve of absorbance vs concentration. Absorbance was read at 540 nm, with absorbance being proportional to the glycosaminoglycan (GAG) content in the samples. GAG content was expressed as a percentage of the tissue weight.

### Histological and immunohistochemical analysis of the organotypic femur cultures

Following μCT analysis, embryonic chick femur samples were dehydrated in a graded series of alcohols and embedded in low-melting point paraffin using an automated Shandon Citadel 2000. 6 μm sections were cut and stained for the nuclear counter-stain Weigert’s hematoxylin, followed by staining with 0.5% alcian blue 8GX for proteoglycan-rich cartilage matrix and 1% Sirius red F3B for collagenous matrix. Sections were also stained with von Kossa for assessing the amount of bone mineralization as previously described [28,29].

Bone samples were analyzed for Type I collagen, Type II collagen, PCNA and STRO-1^+^ expression. In brief, after quenching endogenous peroxidase activity with 3% H_2_O_2_ and blocking with 1% bovine serum albumin (BSA) in 1x PBS, sections were incubated overnight at 4°C with primary antibodies diluted appropriately in 1% BSA in PBS: either LF68 Type I collagen (1:1000), Type II collagen (1:500), PCNA (1:100), or STRO-1^+^ (undiluted culture supernatant obtained from the STRO-1^+^ hybridoma provided by Dr J. Beresford, University of Bath, UK.). An additional hyaluronidase incubation step was included prior to incubation with Type II collagen antibody, and an additional permeabilization step with 0.5% Triton-X was included prior to incubation with PCNA antibody. Following primary antibody incubations, sections were incubated with biotin-conjugated secondary antibodies, diluted appropriately in 1% BSA in PBS: either anti-rabbit IgG (DAKO A/S, Denmark; 1:100) for Type I and Type II collagen, anti-mouse IgG (Sigma, UK, 1:100) for PCNA, or anti-mouse IgM for STRO-1^+^. Visualization of the immune complex involved the avidin-biotin method linked to peroxidase and 3-amino-9-ethylcarbazole (AEC), resulting in a reddish brown reaction product. Sections were counterstained for light green and alcian blue and mounted in aqueous CC/Mount (Sigma, UK). No staining was observed in any control sections in which the primary antibody was omitted. IgM isotype controls were run alongside the STRO-1^+^ antibody incubated tissue sections.

Whole slide images of the histological and immunohistochemical stained sections were analyzed using an Olympus BX-51/22 dotSlide microscope and images created using OlyVIA 2.1 software (Olympus Soft Imaging Solutions, GmBH), and by a Carl Zeiss Axiovert 200 microscope with Carl Zeiss Axiovision 3.1 software package used to capture images. The cell number and the ratio of cells expressing the proliferation marker PCNA in immunohistological sections was quantified by CellProfiler image analysis software [30]. Automated cell numbers were assessed in the entire diaphysis and epiphysis regions of imaged sections of cultured embryonic chick femurs. PCNA positive expressed cells were counted separately and were expressed as a ratio of the total cell numbers counted.

### Statistical analysis

In total 12 femurs were used per group treatment. At the end of the experiments 4 femurs cultured were analyzed for μCT analysis, 4 femurs cultured were processed for histological analysis and the final 4 femurs cultured were analysed for GAG. All measurements were calculated and presented as mean ± standard deviation. Statistical analysis was performed using a one-way ANOVA (GraphPad Prism 3.02 software) and differences between experimental groups confirmed using a two-tailed paired Student’s *t* test according to experimental design and considered to be significantly different if P < 0.05. For PCNA cell number analysis of histological sections all measurements were calculated as mean ± standard error of the mean and statistical analyses performed using GraphPad InStat3 v3.06 software. Differences among groups determined by one-way ANOVA with a post-hoc Tukey’s test, and statistical differences were considered to be significant if P < 0.05.

## Results

### μCT bone analyses of the modulatory effects of 1α,25-dihydroxyvitamin D_3_ on embryonic femur development

**Fig 1:**
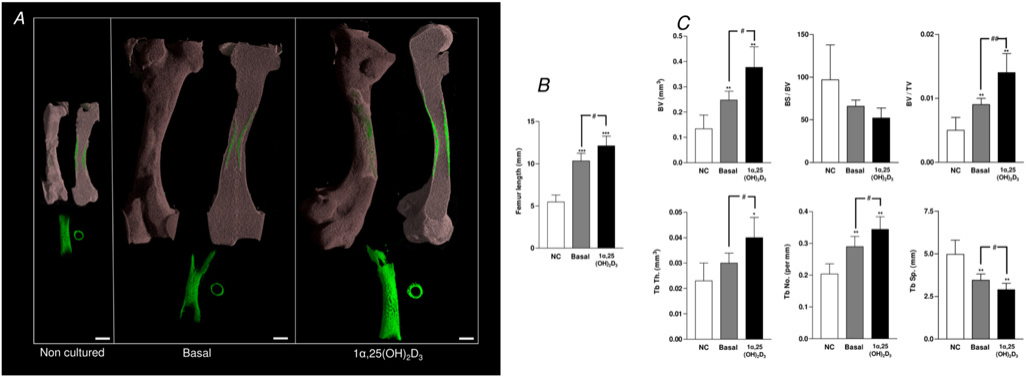
μCT analysis of organotypic cultured embryonic femurs in basal and 1,25(OH)2D3 conditions. A, μCT images (whole femur tissue; saggital sections; segmented mineralized bone (green); and cross sectional sections of the central diaphysis region) of the embryonic chick femurs (E11) organotypic cultured in basal and 1α,25(OH)_2_D_3_ media for 10 days. B, Femur lengths ***P < 0.001 increase in femur length vs non-cultured (NC) femurs; ^#^P< 0.05 increase in femur length of 1α,25(OH)_2_D_3_ cultured femurs vs basal cultured femurs. C, μCT morphometric indices of the structure of the E11 embryonic chick femurs either non-cultured (NC) or organotypic cultured in basal and 1α,25(OH)_2_D_3_ media. Values are means ± s.d. (n = 4 femurs per group) *P < 0.05; **P < 0.01 increase or decrease in μCT bone morphometric indices of basal and 1α,25(OH)_2_D_3_ cultured femurs compared to non-cultured (NC) femurs. #P< 0.05; ##P< 0.01 increase/decrease in μCT bone morphometric indices of 1α,25(OH)_2_D_3_ compared to basal cultured embryonic femurs. (Scale bar = 1mm).

**Fig 2:**
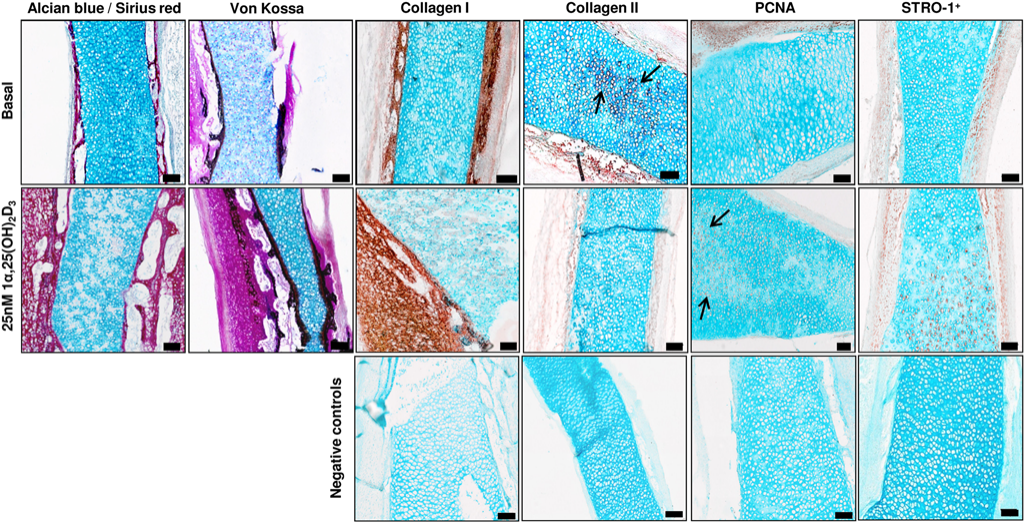
Histological analysis of E11 embryonic chick femurs organotypic cultures. E11 femurs cultured in basal and 1α,25(OH)_2_D_3_ supplemented media for 10 days were analyzed for alcian blue/Sirius red, von Kossa-mineralization, expression of collagen Type I & II (arrows), the proliferation marker PCNA (arrows) and STRO-1^+^. (Scale bar = 100μm). All representative images depict the mid-diaphyseal region of the femur.

**Fig 3:**
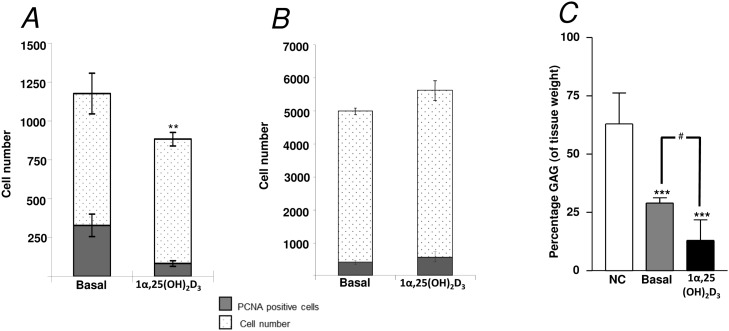
Analysis of diaphyseal and epiphyseal cell proliferation and glycosaminoglycan content of E11 organotypic cultured femurs. *A*, diaphyseal and *B*, epiphyseal cell proliferation data for 1α,25(OH)_2_D_3_ treated femurs (E11) **P < 0.01. *C*, Glycosaminoglycan (GAG) content (expressed as percentage of tissue weight) of embryonic chick femurs, either non-cultured (NC) or cultured for 10 days in basal media, alone or supplemented with 25 nM 1α,25(OH)_2_D_3_. ***P < 0.001 reduced GAG content compared to non-cultured femurs, ^#^ *P* < 0.05 reduced GAG content of 1α,25(OH)_2_D_3_ cultured femurs compared to basal cultured femurs.

The addition of 1α,25(OH)_2_D_3_ (25 nM) to E11 embryonic femur organotypic cultures resulted in a significant increase in the length of the femur (Fig. 1*A* and *B*) as assessed by μCT. μCT confirmed enhanced bone formation as assessed by increased BV, BV/TV, Tb.Th, Tb.No and reduced Tb.Sp in direct contrast to non-cultured and basal cultured embryonic femurs (Fig. 1*C*). The significant augmentation of bone formation indices is correlated with an increase in Sirius red/von Kossa staining and Type I collagen deposition/expression, reduced alcian blue staining and Type II collagen deposition and an increase in STRO-1^+^ expression (Fig. 2, S1 Fig). 1α,25(OH)_2_D_3_ reduced the proliferation of the diaphyseal chondrocytes evidenced by reduced expression of PCNA positive cells compared to the total number of cells (Fig. 3*A*). No significant differences in proliferation were observed in the epiphyseal regions of the femurs (Fig. 3*B*). Furthermore, the exposure of the embryonic chick femurs to 1α,25(OH)_2_D_3_ significantly reduced the GAG content in comparison to basal cultured and non-cultured femurs (Fig. 3*C*).

### μCT bone analyses of the modulatory effects of TGF-β3 on embryonic femur development

**Fig 4:**
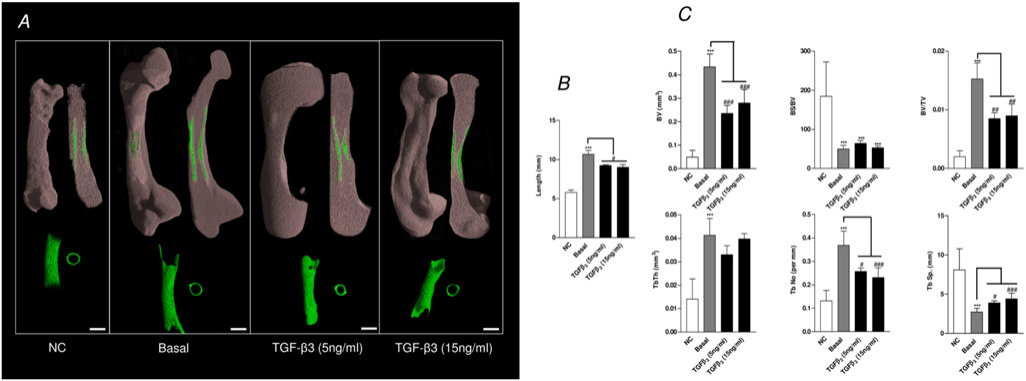
μCT analysis of organotypic cultured embryonic femurs (E11) in basal and TGF-β3 conditions. *A*, μCT images (whole femur tissue; saggital sections; segmented mineralized bone (green); and cross sectional sections of the central diaphysis region) of the embryonic chick femurs organotypic cultured in basal and TGF-β3 (5ng/ml and 15ng/ml) for 10 days. *B*, Femur lengths. ***P < 0.001 increase in basal cultured femur lengths compared to non-cultured (NC) femurs; ^#^ *P* < 0.05 decrease in TGF-β3 cultured femur length compared to basal cultured femurs. *C*, μCT morphometric indices of the structure of the E11 embryonic chick femurs either non-cultured (NC) or organotypic cultured in basal and TGF-β3 media for 10 days. Values are means ± s.d. (n = 4 femurs per group) ***P < 0.01 increase μCT bone morphometric indices of non-cultured embryonic chick femurs compared to basal cultured femurs. ***P < 0.01 increase in μCT bone morphometric indices of basal cultured femurs compared to non-cultured femurs ^#^ *P* < 0.05, ^###^ *P* < 0.001 increase/decrease in μCT bone morphometric indices of TGF-β3 (5 ng/mL and 15 ng/ml) cultured embryonic femurs compared to basal cultured embryonic femurs. (Scale bar = 1mm).

**Fig 5:**
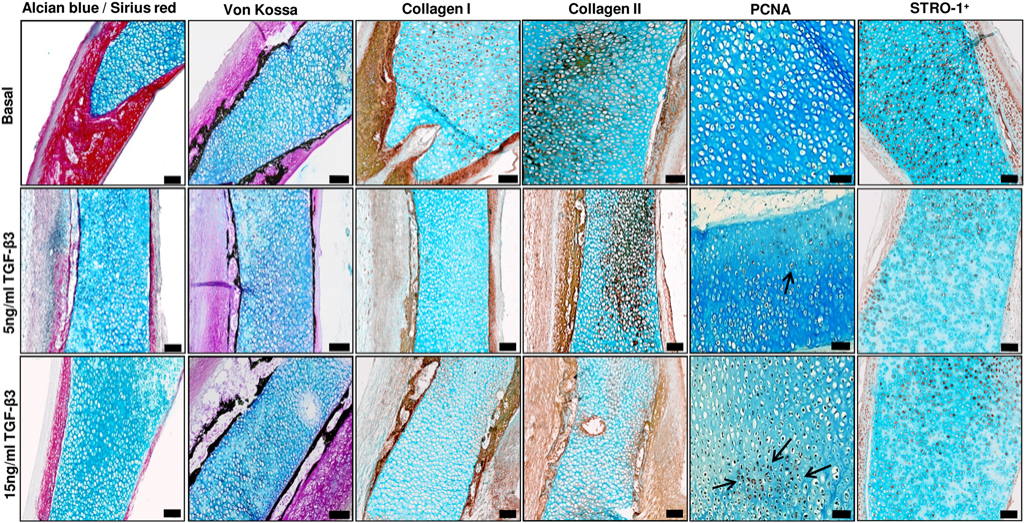
Histological analysis of TGF-β3 effects on E11 embryonic chick femurs organotypic cultures. Histological analysis of E11 embryonic chick femurs organotypic cultured in basal and basal media containing TGF-β3 (5 ng/mL and 15 ng/mL) for alcian blue/Sirius red, von Kossa, expression of collagen Type I & II, the proliferation marker PCNA (arrows) and STRO-1^+^ (scale bar = 100μm). All representative images depict the mid-diaphyseal region of the femur.

**Fig 6:**
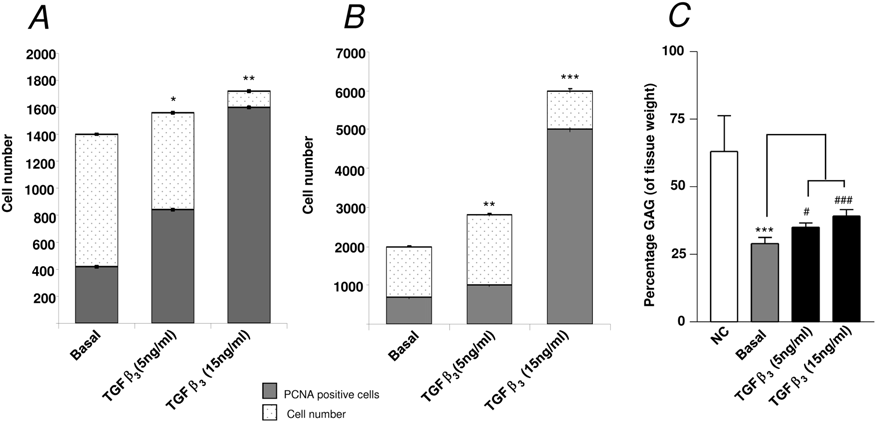
Analysis of diaphyseal and epiphyseal cell proliferation and glycosaminoglycan content of E11 organotypic cultured femurs. *A*, diaphyseal and *B*, epiphyseal cell proliferation data for TGF-β3 treated femurs (E11) *P < 0.05; **P < 0.01; ***P < 0.001. *C*, Glycosaminoglycan (GAG) content (expressed as percentage of tissue weight) of embryonic chick femurs, either non-cultured (NC) or cultured for 10 days in basal media, alone or supplemented with either 5 ng/mL or 15 ng/mL of TGF-β3. ***P < 0.001 reduced GAG content compared to non-cultured femurs, ^#^ *P* < 0.05; ^###^ *P* < 0.001 increased GAG content of 5 ng/mL and 15 ng/mL of TGF-β3 cultured femurs respectively compared to basal cultured femurs.

The addition of the chondrogenic factor TGF-β3 (5 ng/mL and 15 ng/mL) resulted in a statistically significant effect on the bone development of the organotypic cultured femurs. TGF-β3 significantly reduced the growth of the organotypic cultured femurs (E11) compared to control basal femurs (Figs. 4*A* and *B*). In addition, μCT analysis demonstrated that TGF-β3 reduced bone morphometric indices of BV, BV/TV, Tb.No, and increased Tb.Sp within these femurs compared to the basal control femurs (Fig. 4*C*). These results correlated with the data obtained from the histological sections which demonstrated a reduction in Sirius red staining, and expression of Type I collagen and STRO-1^+^ in femurs cultured with TGF-β3 (Fig. 5 and S2 Fig) (-ve controls S4 Fig). Furthermore, TGF-β3 increased alcian blue staining, and expression of Type II collagen and PCNA in the cultured femurs (Fig. 5 and S2 Fig) (-ve controls S4 Fig). TGF-β3 enhanced chondrocyte proliferation within the diaphyseal (Fig. 6*A*) and the epiphyseal (Fig. 6*B*) regions of the femur as evidenced by increased PCNA positive cells compared to the total number of cells. The addition of TGF-β3 to the cultured femurs significantly increased the GAG content compared to the basal cultured femurs (Fig. 6*C*).

**Fig 7:**
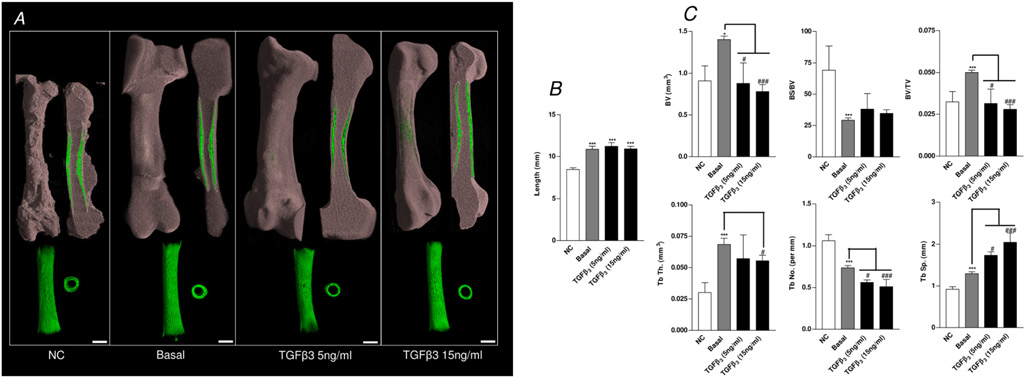
μCT analysis of organotypic cultured embryonic femurs (E13) in basal and TGF-β3 conditions. *A*, μCT images (whole femur tissue; saggital sections; segmented mineralized bone (green); and cross sectional sections of the central diaphysis region) of the embryonic chick femurs (E13) organotypic cultured in basal and TGF-β3 (5ng/ml and 15ng/ml) for 10 days. *B*, Femur lengths; Values are means ± s.d. (n = 4 femurs per group) ***P < 0.001 increase in femur length of basal and TGF-β3 organotypic cultured femurs compared to non-cultured femurs. *C*, μCT morphometric indices of the structure of the E13 embryonic chick femurs either non-cultured (NC) or organotypic cultured in basal and TGF-β3 media for 10 days. Values are means ± s.d. (n = 4 femurs per group) *P < 0.05 ***P < 0.001 increase/decrease in μCT bone morphometric indices of non-cultured embryonic chick femurs compared to basal organotypic cultured femurs. ^#^ *P* < 0.05, ^###^ *P* < 0.001 increase/decrease in μCT bone morphometric indices of TGF-β3 (5 ng/mL and 15 ng/mL) cultured embryonic femurs compared to basal cultured embryonic femurs. (Scale bar = 1mm).

**Fig 8:**
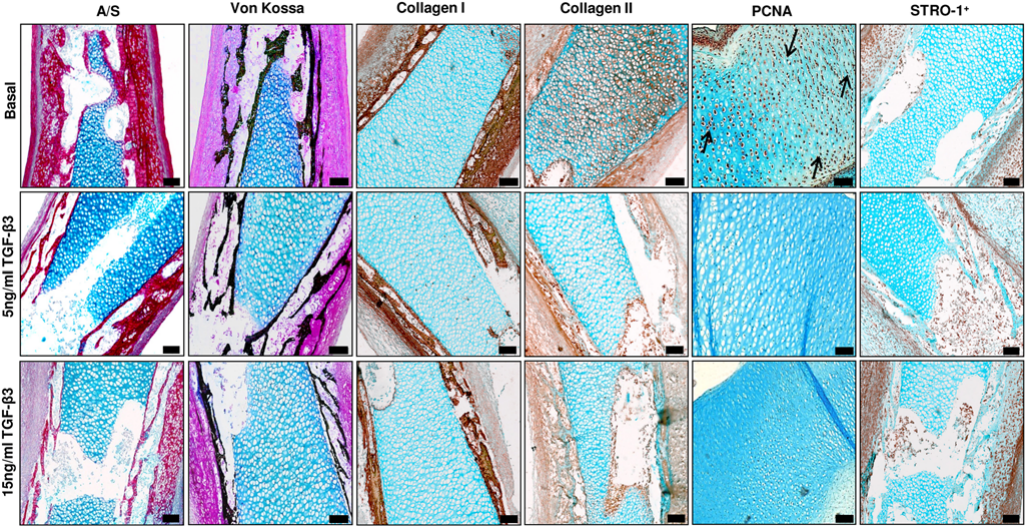
Histological analysis of TGF-β3 effects on E13 embryonic chick femurs organotypic cultures. Histological analysis of the mid-diaphyseal region of organotypic cultured embryonic femurs (E13) in basal and TGF-β3 conditions (scale bar = 100μm). All representative images depict the mid-diaphyseal region of the femur.

**Fig 9:**
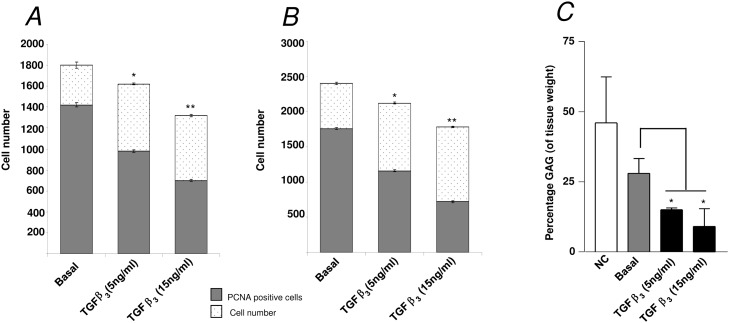
Analysis of diaphyseal and epiphyseal cell proliferation and glycosaminoglycan content of E13 organotypic cultured femurs. *A*, diaphyseal and *B*, epiphyseal cell proliferation data for TGF-β3 treated femurs (E13); *P < 0.05; **P < 0.01. *C*, Glycosaminoglycan (GAG) content (expressed as percentage of tissue weight) of embryonic chick femurs, either non-cultured (NC) or cultured for 10 days in basal media, alone or supplemented with either 5 ng/mL or 15 ng/mL of TGF-β3. GAG content was significantly reduced in both groups of organotypic cultured femurs with TGF-β3 compared to basal cultured femurs (*P < 0.05). Values are mean ± s.d. (n = 4 femurs per group).

In studies using the more developed E13 embryonic femurs, addition of TGF-β3 resulted in a modest effect on growth (Fig. 7*A* and *B*) and bone structure indices with reduced levels of BV, BV/TV, Tb.No, and increased Tb.Sp within these femurs compared to the basal control femurs. Interestingly, 15 ng/mL TGF-β3 significantly reduced the levels of Tb.Th compared to the basal cultured femurs which was not observed in the E11 femurs (Fig. 7*C*). Histological examination of sections from the femurs demonstrated reduced collagen as determined by Sirius red staining, reduced Type I and II collagen expression, reduced mineralization, and reduced cell proliferation within the hypertrophic chondrocytes (Fig. 8 and S3 Fig) (-ve controls S4 Fig). No observable differences were detected in the expression of STRO-1^+^ between the groups except for the femurs cultured in the presence of 15 ng/mL TGF-β3 which displayed a thicker bone collar containing cells positive for the expression of STRO-1^+^ (Fig. 8 and S3 Fig). TGF-β3 decreased the chondrocyte proliferation of the diaphyseal (Fig. 9*A*) and the epiphyseal (Fig. 9*B*) regions of the femur as indicated by reduced numbers of PCNA positive cells compared to the total number of cells. In contrast to the E11 embryonic cultured femurs the addition of TGF-β3 to E13 embryonic cultured femurs significantly reduced the levels of GAG compared to the femurs cultured in basal conditions (Fig. 9*C*).

## Discussion

In recent years advances have been made in identifying embryonic and adult stem cell populations that can differentiate into the appropriate cell lineages capable of contributing to the engineering of complex tissue and organs [31,32]. However, to date, the developmental cues central in the guidance of stem and progenitor cell populations in multi uniform culture systems and the appropriate stimuli for 3D tissue engineering remains limited. Elucidation of the complex cellular interactions, temporal and spatial coordination of factors to produce a functional skeletal structure is a central challenge. Previously, using the organotypic embryonic chick femur culturing system, the modulation of the femur growth and development can be affected by simple changes in growth medium or the addition of a single growth factor to modify culture conditions [28,29] providing a robust system to evaluate the developmental process *in situ*.

In this study we demonstrate the different modulatory effects of the osteotropic agents 1α,25(OH)_2_D_3_ and TGF-β3 on bone femoral growth and development in an *ex vivo* organ culture system. The importance and role of 1α,25(OH)_2_D_3_ in the development and maintenance of the skeleton has been extensively characterized through studies assessing the consequences of nutritional imbalance or limited exposure to sunlight [33,34]. Indeed, in vitamin-D deficient chicken embryos reduced mineralization were found in the tibiae bones with enhanced osteoid regions, reduced organ functions and increased mortality rates [35]. Moreover, even in the early developmental years of the embryo the effects of 1α,25(OH)_2_D_3_ deficiency can have profound effects on skeletal development [12,13].

The current studies highlight the augmentation of bone structural morphometric indices when organotypic embryonic chick femurs were cultured in the presence of 1α,25(OH)_2_D_3_. We found that supplementation of 1α,25(OH)_2_D_3_ (25 nM) to the organotypic femur cultures increased the Bone Volume, Bone Volume/Tissue Volume ratio, Trabecular Thickness and Trabecular Number together with a reduction of Trabecular Spacing. Even the length of the femurs increased in these culture conditions compared to the control femurs. Additionally, 1α,25(OH)_2_D_3_ altered the levels of osteogenesis and chondrogenesis, increased cell proliferation and enhanced the expression of the mesenchymal stem cell marker, STRO-1^+^. The direct osteogenic effect of 1α,25(OH)_2_D_3_ observed on the embryonic chick femur correlated well with previous publications in the literature [1,4,36,37].

Unfortunately, the effect of vitamin D3 is not that clear cut in bone development. Mice born from mothers with vitamin D receptor deficiency display a normal skeletal development at birth but bone defects only occur at weaning [38]. Additionally, chondrocytes in mice with deleted vitamin D receptor or 1-a-hydroxylase have a small effect on the development of the skeleton with an enlargement of the growth plate due to the reduced levels of vascular invasion and active osteoclasts [39]. Moreover vitamin D receptor deletion in mouse osteoblasts/osteocytes has no effect on bone mass [40] whereas specific deletion of this receptor in osteoblasts results in elevated bone mass [41]. Serum 25-hydroxyvitamin D [25(OH)D], 1α,25-dihydroxyvitamin D [1α,25(OH)_2_D_3_], are the predominant metabolites used for the assessment of vitamin D status and evaluation of vitamin D metabolism. The major circulating metabolite, 25(OH)D, is generally considered the approach to define nutritional vitamin D status although there is a debate, as to the requisite healthy minimum level of circulation 25(OH)D, where the active metabolite is 1α,25(OH)_2_D_3_ is responsible for most actions of vitamin D. Recent data using various biomarkers such as intact parathyroid hormone (PTH), intestinal calcium absorption, and skeletal density measurements suggest that the adequate healthy range for 25(OH)D and 1α,25(OH)_2_D_3_ is 20–150 nM and 50–150 pM respectively [42]. However, the evidence highlight the importance of the correct levels of an active vitamin D metabolite and demonstrates the potential of the organotypic model developed in conjunction with the use of μCT analysis to determine significant changes in bone development particularly using small dose ranges of 1α,25(OH)_2_D_3._ This is currently under investigation in our labs now. It must be also stated we have only concentrated on 1α,25(OH)_2_D_3_ but there are various other metabolites that are associated with the vitamin D_3_ endocrine system. One of these other active metabolites is (24R), 24,25-dihydroxyvitamin D_3_ that is produced by osteoblasts in the bone niche increases osteogenic differentiation of human mesenchymal stem cells and has a pivotal role in skeletal development [43–45].

The mechanistic effect of 1α,25(OH)_2_D_3_ in our studies and in previous *in vivo* studies appears to be very complex. The E11 femurs in our studies have very few osteoclasts (predominantly preosteoclasts residing in the periosteum) to induce a remodelling effect by the administration of 1α,25(OH)_2_D_3_ and hence transposing its effect to bone formation. Additionally, 1α,25(OH)_2_D_3_ could dampen down RANKL expression in MSC and osteoblasts hence reducing the effects of osteoclast on bone formation as recently evidenced by Harada et al, 2012 [46]. Indeed, a lack of 1α,25(OH)_2_D_3_ during development could predispose MSC development into osteoblast with higher RANKL receptor expression. If prolonged reduction in 1α,25(OH)_2_D_3_ levels during development persists an epigenetic effect could result in the MSC or osteoblast having higher levels of RANKL expression leading to bone diseases such as rickets or osteoporosis in later life. Further work would be required to elucidate this outcome.

Although the well characterised fundamental role of 1α,25(OH)_2_D_3_ is in calcium metabolism and bone formation it has been found to also play a role in cartilage modelling and hence endochondral bone growth. 1α,25(OH)_2_D_3_ has the ability to regulate chondrocyte mRNA, TGF-beta expression and TGF-beta secretion in the growth plate of chicks [47] and can directly increase the maturation of chondrocytes subsequently leading to the calcification of the developing chick cartilage [48].

In contrast to the observed effects of 1α,25(OH)_2_D_3_ on skeletal development of the organotypic cultured femurs, supplementation of TGF-β3 significantly inhibited bone formation in both E11 and E13 organotypic cultured embryonic femurs. On closer examination, the levels of detected glycosaminoglycans were elevated with the addition of TGF-β3 to the cultures of E11 staged femurs. In contrast, the opposite effect was observed when the TGF-β3 was added to cultures of E13 staged femurs, indicating the short time frame the growth factor has on its effect on the growth and development of the femur. Previous studies have identified that TGF-β3 can reduce the osteogenic effect in human MSCs [7] and that the development and calcification of bone correlates to a reduction of glycosaminoglycan content of the bone organo matrix [49]. Our results demonstrate that TGF-β3 has an inhibitory effect in the developing bone and hence may have a role to play in reducing the development of ectopic bone in tissue engineering paradigms for tendon or articular cartilage repair. Combined growth factors, TGF-β3 and BMP-2 can enhance the differentiation of cartilage of chick limbs through independent mechanisms that regulate the mesenchymal cells through endochondral ossification [50]. Interestingly, it has been found that differing signaling mechanisms are used in the chondrogenic differentiation of fetal and adult MSCs. TGF-β3 induced chondrogenesis of adult MSCs but failed to induce chondrogenesis in fetal MSCs *in vitro*. However, addition of BMP-2 to the TGF-β3 human fetal cultures induced chondrogenesis similar to the human adult MSCs and TGF-β3 [51]. Similar to our femur cultures the small differences of adding an extra growth factor or osteogenic factor can influence the chondrogenc differentiation of MSCs which could influence the way cartilage is repaired or even crucially the role in actually restricting the onset of osteoarthritis. Analysis by Tang et al, 2009 [25] of recent studies using TGF-β3 and MSCs has indicated that TGF-β3 in a concentration and time dependent mode modulates human MSC chondrogenesis *in vitro* [25]. In translational *in vivo* applications ovine MSCs stimulated by TGF-β3 encapsulated in a chitosan scaffold could successfully repair the partial thickness lesion of the hyaline cartilage two months post implantation, where the implanted scaffold/cells demonstrated very good integration with the host cartilage [52]. Furthermore, in rabbit studies where the articular surface of the proximal humeral condyles were excised and replaced with poly-epsilon-caprolactone and hydroxyapatite bioscaffolds infused with TGF-β3 the investigators were able to demonstrate after 4 months regeneration of the articular surface of the synovial joint without implantation of cells [53].

The current studies illustrate the importance of analyzing the effects of individual factors *in situ* in the femoral organotypic model within an accompanying complex cell population and architecture to identify and understand the changes in organ/tissue growth, development and function. Moreover, at the level of rudiment organ bone culture, 1α,25(OH)_2_D_3_ and TGF-β3 exerted dramatic and concentration dependent effects on the development of the femoral bone. Understanding and applying the principle mechanisms that orchestrate fetal skeletal development can only better inform reparative approaches [27]. For example it has been shown that the use of complex bioreactors (BioDomes) to enhance tissue regrowth of digits *in vivo* by providing the necessary environmental cues including electrical stimulation can activate stem cells and temporospatial tissue reorganisation and hence tissue regeneration [54]. The combination of cells, factors and biocomposites will have important implications in particular the need to apply relevant spatial, temporal and concentration controlled release of components that are aligned for fully functional tissue/organ development. This will enhance the regenerative properties of stem cells within the 3D tissue/organ structure. Thus, collaborative investigations across the fields of tissue engineering and developmental biology will aid our understanding of skeletal tissue development and provide novel regenerative technologies. Furthermore, elucidating the microenvironmental niches of embryonic skeletal development may lead to better stem cell translational therapy, which currently, at best, has seen limited progression to the clinic [26].

## Conclusions

Fundamental to clinical bone regeneration and repair is the appropriate creation of composites that mimic the release profile of factors stimulating growth and repair to enhance the capability of implanted cell progenitor(s) to maximize their potential. Bone development and the recapitulation in organ systems particularly the incorporation of a functional vasculature will aid future regenerative medicine strategies to address the complex nature and timing of the growth and repair of organ/tissue systems. μCT together with molecular mapping and cell fate tracing offer new approaches to understand skeletal development and to inform regenerative programs in producing better outcomes for bone repair and regeneration.

## Supporting Information

S1 Fig Histological analysis of 1α,25(OH)_2_D_3_ effects on E11 embryonic chick femurs organotypic cultures. E11 femurs cultured in basal and 1α,25(OH)_2_D_3_ supplemented media were analyzed for alcian blue/Sirius red, von Kossa-mineralization, expression of collagen Type I & II, the proliferation marker PCNA and STRO-1^+^ (scale bar = 500 μm).
(TIF)

S2 Fig Histological analysis of TGF-β3 effects on E11 embryonic chick femurs organotypic cultures. Histological analysis of embryonic chick femurs organotypic cultured in basal and basal media containing TGF-β3 (5 ng/mL and 15 ng/mL) for alcian blue/Sirius red, von Kossa, expression of collagen Type I & II, the proliferation marker PCNA and STRO-1^+^ (scale bar = 500 μm).
(TIF)

S3 Fig Histological analysis of TGF-β3 effects on E13 embryonic chick femurs organotypic cultures. Histological analysis of embryonic chick femurs (E13) organotypic cultured in basal and basal media containing TGF-β3 (5 ng/mL and 15 ng/ml) for alcian blue/Sirius red, von Kossa, expression of collagen Type I & II, the proliferation marker PCNA and STRO-1^+^ (scale bar = 500 μm).
(TIF)

S4 Fig Immunohistochemistry negative control images. Representative low and high powered images of type I collagen, type II collagen II, PCNA and STRO-1^+^ immunohistochemistry negative controls (primary antibody omission) of embryonic femurs organotypic cultured for 10 days. Top row E11 femurs cultured with 1α,25(OH)_2_D_3_, middle row E11 femurs cultured with TGF-β3, bottom row E13 femurs cultured with TGF-β3. Scale bar top two rows = 100μm; bottom row = 500 μm.
(TIF)

## References

[1] Bagi CM, Miller SC (1992) Dose-related effects of 1, 25-dihydroxyvitamin D3 on growth, modeling, and morphology of fetal mouse metatarsals cultured in serum-free medium. Journal of Bone and Mineral Research 7: 29–40. 1549956 10.1002/jbmr.5650070106

[2] Kovacs CS (2011) Bone development in the fetus and neonate: role of the calciotropic hormones. Current osteoporosis reports 9: 274–283. 10.1007/s11914-011-0073-0 21904825

[3] Adams JS, Hewison M (2010) Update in vitamin D. The Journal of Clinical Endocrinology & Metabolism 95: 471–478. 20133466 10.1210/jc.2009-1773 PMC2840860

[4] Lanske B, Razzaque MS (2007) Vitamin D and aging: old concepts and new insights. The Journal of nutritional biochemistry 18: 771–777. 17531460 10.1016/j.jnutbio.2007.02.002 PMC2776629

[5] Cheah FS, Winkler C, Jabs EW, Chong SS (2010) *tgfβ3* regulation of chondrogenesis and osteogenesis in zebrafish is mediated through formation and survival of a subpopulation of the cranial neural crest. Mechanisms of development 127: 329–344. 10.1016/j.mod.2010.04.003 20406684

[6] Bouffi C, Thomas O, Bony C, Giteau A, Venier-Julienne M-C, et al (2010) The role of pharmacologically active microcarriers releasing TGF-β3 in cartilage formation *in vivo* by mesenchymal stem cells. Biomaterials 31: 6485–6493. 10.1016/j.biomaterials.2010.05.013 20570347

[7] Moioli EK, Hong L, Mao JJ (2007) Inhibition of osteogenic differentiation of human mesenchymal stem cells. Wound repair and regeneration 15: 413–421. 17537129 10.1111/j.1524-475X.2007.00244.x PMC4035040

[8] DeLuca HF (2004) Overview of general physiologic features and functions of vitamin D. The American journal of clinical nutrition 80: 1689S–1696S. 15585789 10.1093/ajcn/80.6.1689S

[9] Woeckel V, Bruedigam C, Koedam M, Chiba H, van der Eerden B, et al (2013) 1α, 25-dihydroxyvitamin D3 and rosiglitazone synergistically enhance osteoblast-mediated mineralization. Gene 512: 438–443. 10.1016/j.gene.2012.07.051 22967709

[10] Endo H, Kiyoki M, Kawashima K, Naruchi T, Hashimoto Y (1980) Vitamin D3 metabolites and PTH synergistically stimulate bone formation of chick embryonic femur in vitro. 10.1038/286262a0 7402314

[11] Dror DK (2011) Vitamin D status during pregnancy: maternal, fetal, and postnatal outcomes. Current Opinion in Obstetrics and Gynecology 23: 422–426. 10.1097/GCO.0b013e32834cb791 21986726

[12] Harvey N, Cooper C (2004) The developmental origins of osteoporotic fracture. British Menopause Society Journal 10: 14–29. 15107206 10.1258/136218004322986726

[13] Hewison M, Adams JS (2010) Vitamin D insufficiency and skeletal development in utero. Journal of Bone and Mineral Research 25: 11–13. 10.1002/jbmr.2 20091930 PMC4847429

[14] Davies JH, Reed JM, Blake E, Priesemann M, Jackson AA, et al (2011) Epidemiology of vitamin D deficiency in children presenting to a pediatric orthopaedic service in the UK. Journal of Pediatric Orthopaedics 31: 798–802. 10.1097/BPO.0b013e31822f1af1 21926880

[15] Mehlhorn A, Schmal H, Kaiser S, Lepski G, Finkenzeller G, et al (2006) Mesenchymal stem cells maintain TGF-β-mediated chondrogenic phenotype in alginate bead culture. Tissue engineering 12: 1393–1403. 16846338 10.1089/ten.2006.12.1393

[16] Ma HL, Hung SC, Lin SY, Chen YL, Lo WH (2003) Chondrogenesis of human mesenchymal stem cells encapsulated in alginate beads. Journal of Biomedical Materials Research Part A 64: 273–281. 12522814 10.1002/jbm.a.10370

[17] Park JS, Woo DG, Yang HN, Na K, Park K-H (2009) Transforming growth factor beta-3 bound with sulfate polysaccharide in synthetic extracellular matrix enhanced the biological activities for neocartilage formation in vivo. Journal of Biomedical Materials Research Part A 91A: 408–415. 10.1002/jbm.a.32271 18985789

[18] Matsunaga S, Yamamoto T, Fukumura K (1999) Temporal and spatial expressions of transforming growth factor-betas and their receptors in epiphyseal growth plate. International journal of oncology 14: 1063–1070. 10339658 10.3892/ijo.14.6.1063

[19] Thorp BH, Anderson I, Jakowlew SB (1992) Transforming growth factor-beta 1,-beta 2 and-beta 3 in cartilage and bone cells during endochondral ossification in the chick. Development 114: 907–911. 1618152 10.1242/dev.114.4.907

[20] Thorp B, Jakowlew S (1994) Altered localisation of transforming growth factor-β3 during endochondral ossification in rachitic chicks. Bone 15: 59–64. 8024853 10.1016/8756-3282(94)90892-3

[21] Opperman LA, Adab K, Gakunga PT (2000) Transforming growth factor-2β and TGF-β 3 regulate fetal rat cranial suture morphogenesis by regulating rates of cell proliferation and apoptosis. Developmental Dynamics 219: 237–247. 11002343 10.1002/1097-0177(2000)9999:9999<::AID-DVDY1044>3.0.CO;2-F

[22] Longaker MT (2001) Role of TGF-beta signaling in the regulation of programmed cranial suture fusion. Journal of Craniofacial Surgery 12: 389–390. 11482626 10.1097/00001665-200107000-00016

[23] Poisson E, Sciote JJ, Koepsel R, Cooper GM, Opperman LA, et al. (2009) Transforming growth factor-β isoform expression in the perisutural tissues of craniosynostotic rabbits. 10.1597/02-140.1 15222795

[24] Opperman L, Chhabra A, Cho R, Ogle R (1998) Cranial suture obliteration is induced by removal of transforming growth factor (TGF)-beta 3 activity and prevented by removal of TGF-beta 2 activity from fetal rat calvaria in vitro. Journal of craniofacial genetics and developmental biology 19: 164–173. 10589398

[25] Tang QO, Shakib K, Heliotis M, Tsiridis E, Mantalaris A, et al. (2009) TGF-β3: A potential biological therapy for enhancing chondrogenesis. 10.1517/14712590902936823 19426117

[26] Turner NJ, Keane TJ, Badylak SF (2013) Lessons from developmental biology for regenerative medicine. Birth Defects Research Part C: Embryo Today: Reviews 99: 149–159. 10.1002/bdrc.21040 24078493

[27] Smith EL, Kanczler JM, Oreffo RO (2013) A new take on an old story: chick limb organ culture for skeletal niche development and regenerative medicine evaluation. European cells & materials 26: 91–106. 24027022 10.22203/ecm.v026a07

[28] Kanczler JM, Smith EL, Roberts CA, Oreffo RO (2012) A novel approach for studying the temporal modulation of embryonic skeletal development using organotypic bone cultures and microcomputed tomography. Tissue Engineering Part C: Methods 18: 747–760. 22472170 10.1089/ten.tec.2012.0033 PMC3460619

[29] Smith EL, Kanczler JM, Roberts CA, Oreffo RO (2012) Developmental cues for bone formation from parathyroid hormone and parathyroid hormone-related protein in an ex vivo organotypic culture system of embryonic chick femora. Tissue Engineering Part C: Methods 18: 984–994. 10.1089/ten.TEC.2012.0132 22690868 PMC4014091

[30] Kamentsky L, Jones TR, Fraser A, Bray M-A, Logan DJ, et al (2011) Improved structure, function and compatibility for CellProfiler: modular high-throughput image analysis software. Bioinformatics 27: 1179–1180. 10.1093/bioinformatics/btr095 21349861 PMC3072555

[31] Crisan M, Yap S, Casteilla L, Chen C-W, Corselli M, et al (2008) A perivascular origin for mesenchymal stem cells in multiple human organs. Cell stem cell 3: 301–313. 10.1016/j.stem.2008.07.003 18786417

[32] Pittenger MF, Mackay AM, Beck SC, Jaiswal RK, Douglas R, et al (1999) Multilineage potential of adult human mesenchymal stem cells. science 284: 143–147. 10102814 10.1126/science.284.5411.143

[33] Holick MF (2004) Sunlight and vitamin D for bone health and prevention of autoimmune diseases, cancers, and cardiovascular disease. The American journal of clinical nutrition 80: 1678S–1688S. 15585788 10.1093/ajcn/80.6.1678S

[34] Yoshida T, Stern PH (2012) How vitamin D works on bone. Endocrinology and metabolism clinics of North America 41: 557–569. 10.1016/j.ecl.2012.04.003 22877429

[35] Narbaitz R, Tsang C (1989) Vitamin D deficiency in the chick embryo: effects on prehatching motility and on the growth and differentiation of bones, muscles, and parathyroid glands. Calcified tissue international 44: 348–355. 2496907 10.1007/BF02556316

[36] Beresford J, Gallagher J, Russell R (1986) 1, 25-Dihydroxyvitamin D3 and Human Bone-Derived Cells in Vitro: Effects on Alkaline Phosphatase, Type I Collagen and Proliferation*. Endocrinology 119: 1776–1785. 3489608 10.1210/endo-119-4-1776

[37] Matsumoto T, Igarashi C, Takeuchi Y, Harada S, Kikuchi T, et al (1991) Stimulation by 1, 25-Dihydroxyvitamin D_3_ of in vitro mineralization induced by osteoblast-like MC3T3-E1 cells. Bone 12: 27–32. 2054233 10.1016/8756-3282(91)90051-j

[38] Li YC, Pirro AE, Amling M, Delling G, Baron R, et al (1997) Targeted ablation of the vitamin D receptor: an animal model of vitamin D-dependent rickets type II with alopecia. Proceedings of the National Academy of Sciences 94: 9831–9835. 9275211 10.1073/pnas.94.18.9831 PMC23277

[39] Masuyama R, Stockmans I, Torrekens S, Van Looveren R, Maes C, et al (2006) Vitamin D receptor in chondrocytes promotes osteoclastogenesis and regulates FGF23 production in osteoblasts. The Journal of clinical investigation 116: 3150–3159. 17099775 10.1172/JCI29463 PMC1635166

[40] Lieben L, Masuyama R, Torrekens S, Van Looveren R, Schrooten J, et al (2012) Normocalcemia is maintained in mice under conditions of calcium malabsorption by vitamin D–induced inhibition of bone mineralization. The Journal of clinical investigation 122: 1803–1815. 10.1172/JCI45890 22523068 PMC3336970

[41] Yamamoto Y, Yoshizawa T, Fukuda T, Shirode-Fukuda Y, Yu T, et al (2013) Vitamin D receptor in osteoblasts is a negative regulator of bone mass control. Endocrinology 154: 1008–1020. 10.1210/en.2012-1542 23389957

[42] Lips P (2007) Relative value of 25 (OH) D and 1, 25 (OH) 2D measurements. Journal of Bone and Mineral Research 22: 1668–1671. 17645404 10.1359/jbmr.070716

[43] Howard GA, Turner R, Sherrard D, Baylink DJ (1981) Human bone cells in culture metabolize 25-hydroxyvitamin D3 to 1, 25-dihydroxyvitamin D3 and 24, 25-dihydroxyvitamin D3. Journal of Biological Chemistry 256: 7738–7740. 6973569

[44] St-Arnaud R (2010) CYP24A1-deficient mice as a tool to uncover a biological activity for vitamin D metabolites hydroxylated at position 24. The Journal of steroid biochemistry and molecular biology 121: 254–256. 10.1016/j.jsbmb.2010.02.002 20144713

[45] Curtis KM, Aenlle KK, Roos BA, Howard GA (2014) 24 R, 25-Dihydroxyvitamin D3 Promotes the Osteoblastic Differentiation of Human Mesenchymal Stem Cells. Molecular Endocrinology 28: 644–658. 10.1210/me.2013-1241 24597546 PMC4004781

[46] Harada S, Mizoguchi T, Kobayashi Y, Nakamichi Y, Takeda S, et al (2012) Daily administration of eldecalcitol (ED-71), an active vitamin D analog, increases bone mineral density by suppressing RANKL expression in mouse trabecular bone. Journal of Bone and Mineral Research 27: 461–473. 10.1002/jbmr.555 22052469

[47] Farquharson C, Law A, Seawright E, Burt D, Whitehead C (1996) The expression of transforming growth factor-β by cultured chick growth plate chondrocytes: differential regulation by 1, 25-dihydroxyvitamin D3. Journal of endocrinology 149: 277–285. 8708539 10.1677/joe.0.1490277

[48] Suda S, Takahashi N, Shinki T, Horiuchi N, Yamaguchi A, et al (1985) 1-ALPHA,25-DIHYDROXYVITAMIN-D3 RECEPTORS AND THEIR ACTION IN EMBRYONIC CHICK CHONDROCYTES. Calcified Tissue International 37: 82–90. 2986803 10.1007/BF02557684

[49] Grynpas MD, Hunter GK (1988) Bone mineral and glycosaminoglycans in newborn and mature rabbits. Journal of Bone and Mineral Research 3: 159–164. 3213611 10.1002/jbmr.5650030206

[50] Roark EF, Greer K (1994) Transforming growth factor-β and bone morphogenetic protein-2 act by distinct mechanisms to promote chick limb cartilage differentiation in vitro. Developmental dynamics 200: 103–116. 7919498 10.1002/aja.1002000203

[51] Brady K, Dickinson SC, Guillot PV, Polak J, Blom AW, et al (2013) Human fetal and adult bone marrow-derived mesenchymal stem cells use different signaling pathways for the initiation of chondrogenesis. Stem cells and development 23: 541–554. 10.1089/scd.2013.0301 24172175 PMC3929258

[52] Mrugala D, Bony C, Neves N, Caillot L, Fabre S, et al (2008) Phenotypic and functional characterisation of ovine mesenchymal stem cells: application to a cartilage defect model. Annals of the rheumatic diseases 67: 288–295. 17644536 10.1136/ard.2007.076620

[53] Lee CH, Cook JL, Mendelson A, Moioli EK, Yao H, et al (2010) Regeneration of the articular surface of the rabbit synovial joint by cell homing: a proof of concept study. The Lancet 376: 440–448. 10.1016/S0140-6736(10)60668-X 20692530 PMC4035014

[54] Hechavarria D, Dewilde A, Braunhut S, Levin M, Kaplan DL (2010) BioDome regenerative sleeve for biochemical and biophysical stimulation of tissue regeneration. Medical engineering & physics 32: 1065–1073. 20708956 10.1016/j.medengphy.2010.07.010 PMC2967604

