# Supplementary figures and images for: The Effects of 1α, 25-dihydroxyvitamin D3 and Transforming Growth Factor-β3 on Bone Development in an Ex Vivo Organotypic Culture System of Embryonic Chick Femora
Source: PLoS One. 2015 Apr 2;10(4):e0121653. doi: 10.1371/journal.pone.0121653 (PMC4383569; doi:10.1371/journal.pone.0121653)

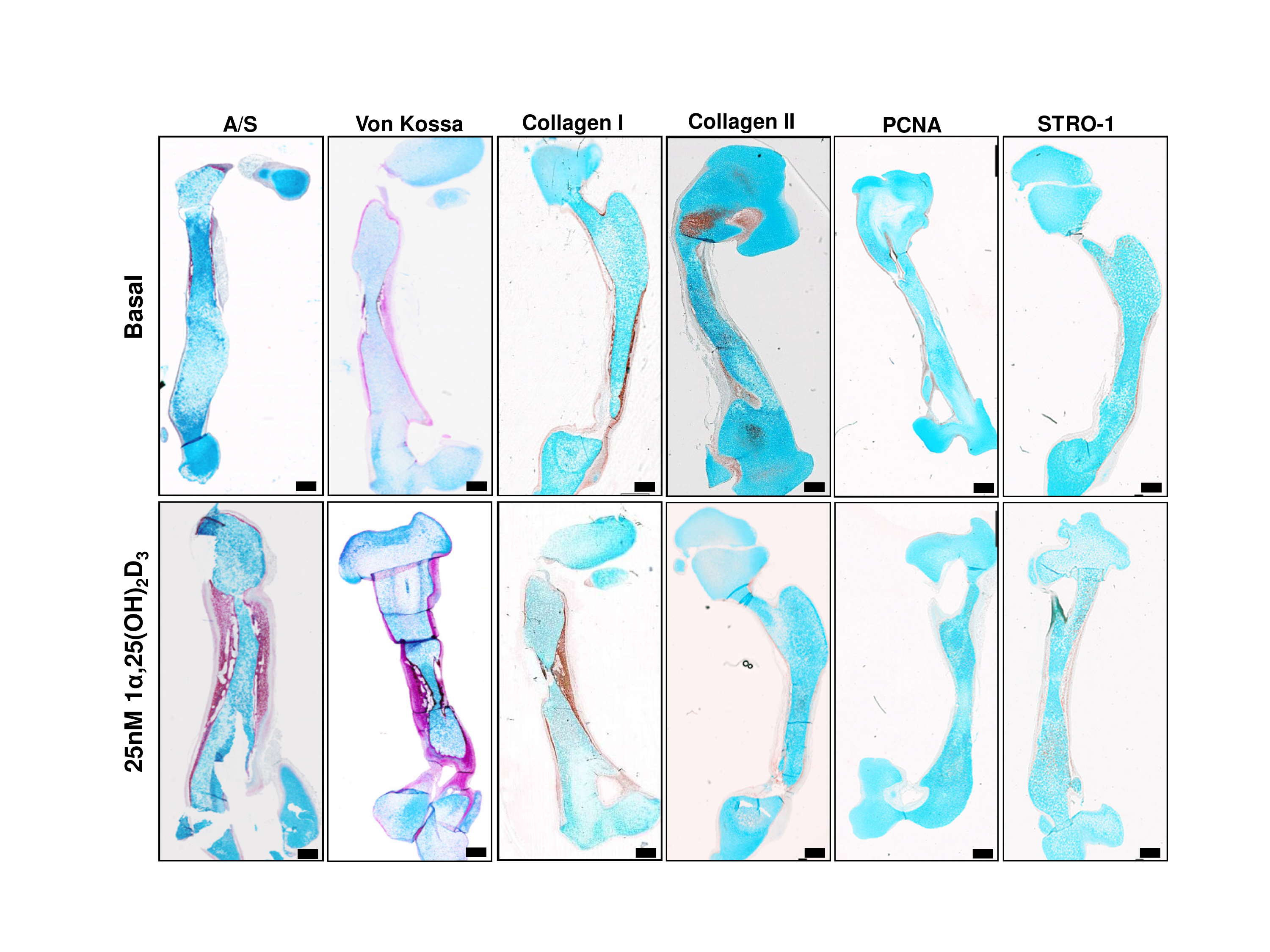

Supplement: S1 Fig — E11 femurs cultured in basal and 1α,25(OH)2D3 supplemented media were analyzed for alcian blue/Sirius red, von Kossa-mineralization, expression of collagen Type I & II, the proliferation marker PCNA and STRO-1+ (scale bar = 500 μm). (TIF) [file pone.0121653.s001.tif]

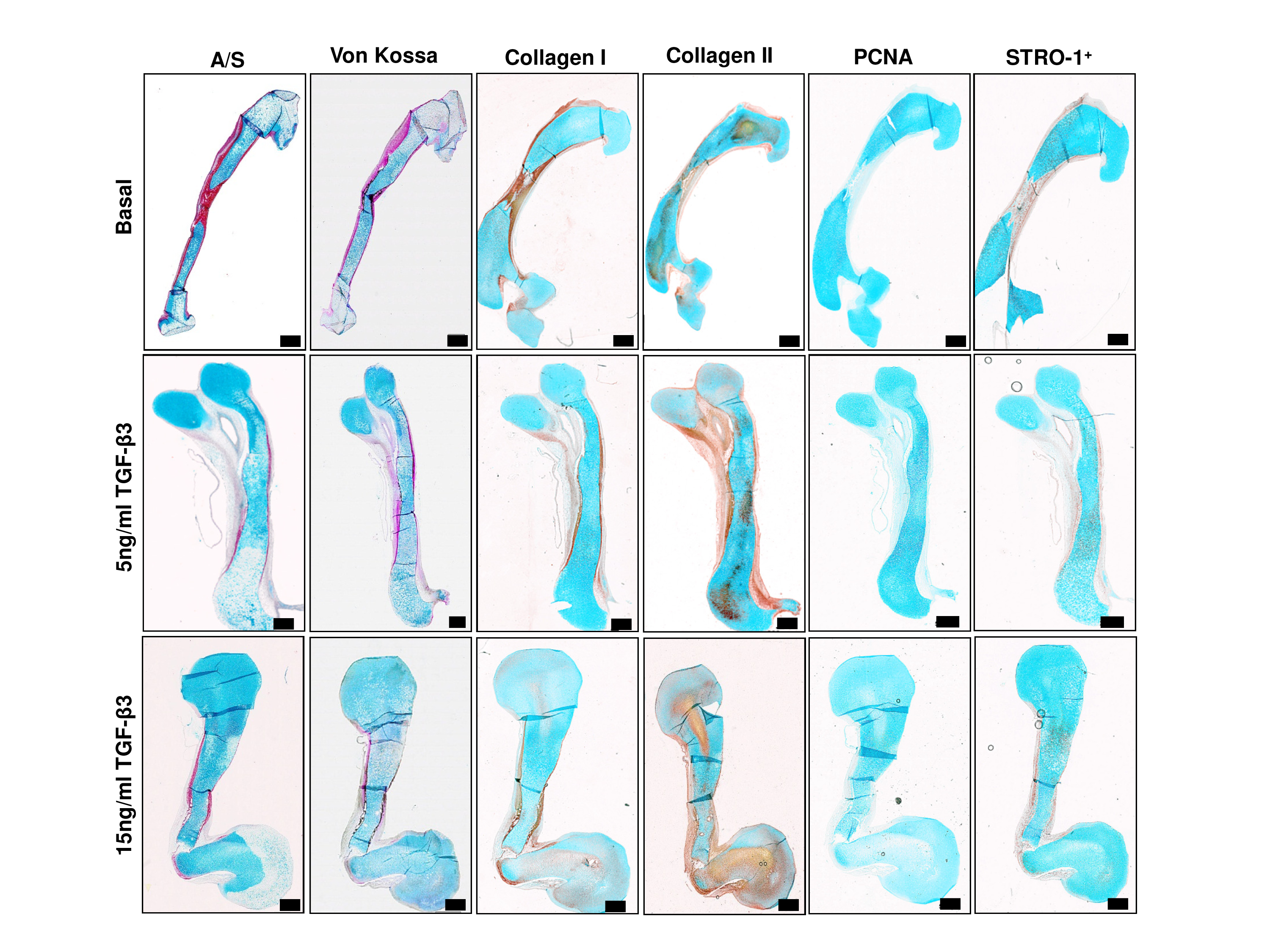

Supplement: S2 Fig — Histological analysis of embryonic chick femurs organotypic cultured in basal and basal media containing TGF-β3 (5 ng/mL and 15 ng/mL) for alcian blue/Sirius red, von Kossa, expression of collagen Type I & II, the proliferation marker PCNA and STRO-1+ (scale bar = 500 μm). (TIF) [file pone.0121653.s002.tif]

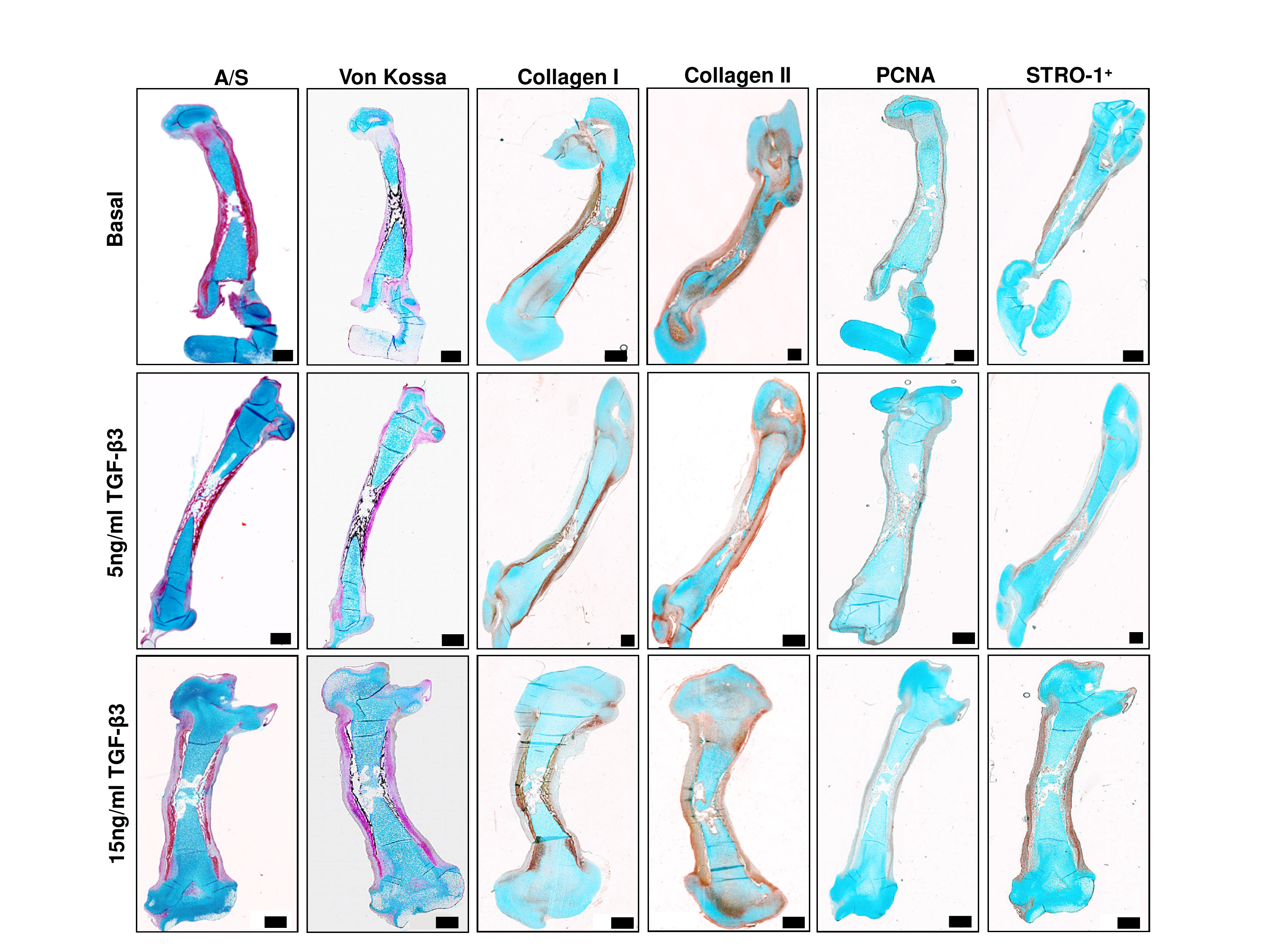

Supplement: S3 Fig — Histological analysis of embryonic chick femurs (E13) organotypic cultured in basal and basal media containing TGF-β3 (5 ng/mL and 15 ng/ml) for alcian blue/Sirius red, von Kossa, expression of collagen Type I & II, the proliferation marker PCNA and STRO-1+ (scale bar = 500 μm). (TIF) [file pone.0121653.s003.tif]

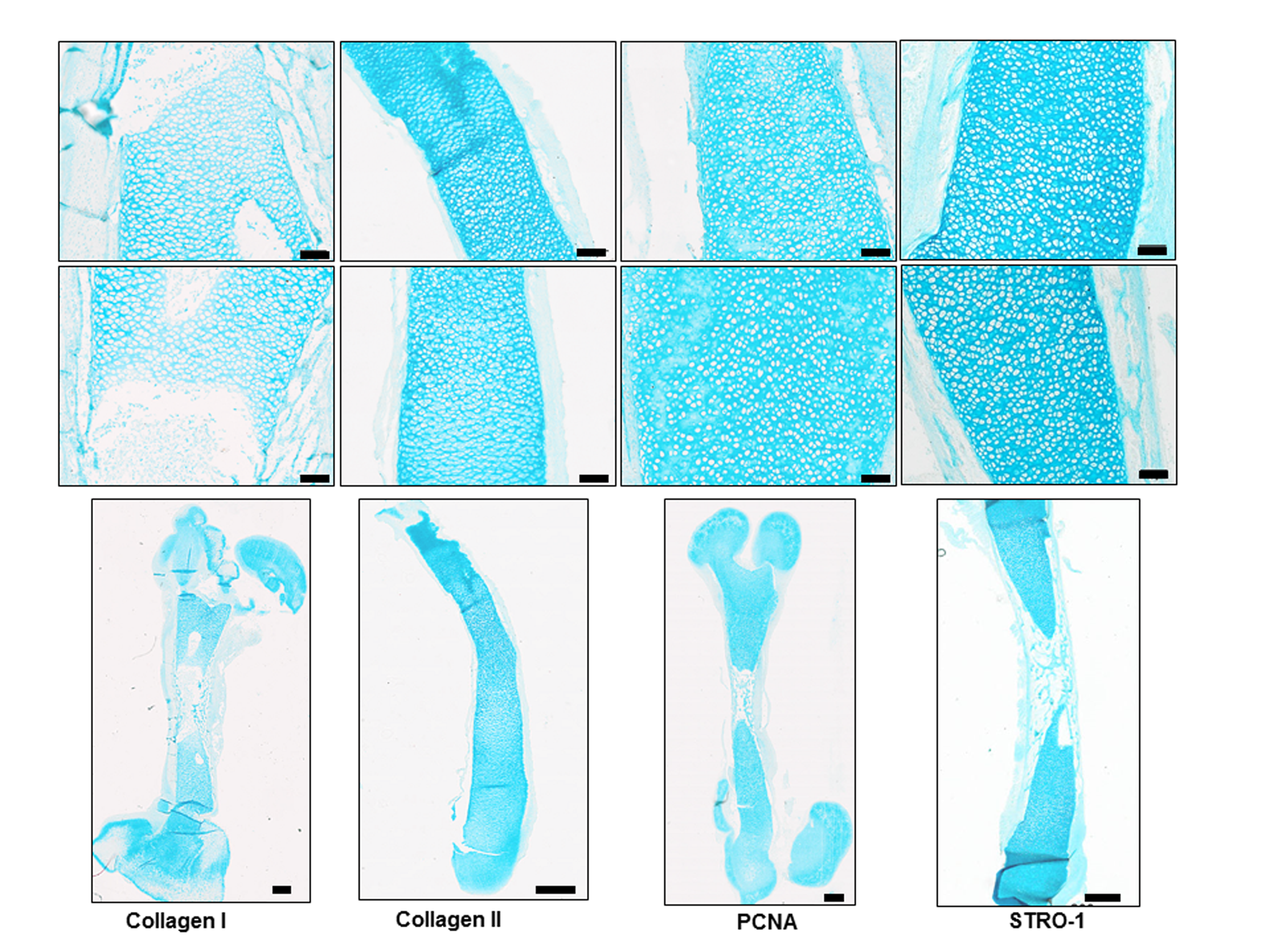

Supplement: S4 Fig — Representative low and high powered images of type I collagen, type II collagen II, PCNA and STRO-1+ immunohistochemistry negative controls (primary antibody omission) of embryonic femurs organotypic cultured for 10 days. Top row E11 femurs cultured with 1α,25(OH)2D3, middle row E11 femurs cultured with TGF-β3, bottom row E13 femurs cultured with TGF-β3. Scale bar top two rows = 100μm; bottom row = 500 μm. (TIF) [file pone.0121653.s004.tif]
